# Comparative Utility of B-type Natriuretic Peptide (BNP) and N-terminal Pro-BNP (NT-proBNP) in the Management of Acute Heart Failure: A Study From a Tertiary Care Hospital in Kashmir, India

**DOI:** 10.7759/cureus.108436

**Published:** 2026-05-07

**Authors:** Javeed A Ganie, Hyder H Lone, Mehraj Ul Islam, Mohd Ismail, Sayima Nargis, Amir Farooq, Mohd Muntazir

**Affiliations:** 1 Department of Medicine, Government Medical College Srinagar, Srinagar, IND; 2 Department of Cardiology, Sher I Kashmir Institute of Medical Sciences, Srinagar, IND; 3 Department of Interventional Nephrology, Sir Gangaram Hospital, New Delhi, IND; 4 Department of Oncoanaesthesia, Delhi Cancer Institute, New Delhi, IND

**Keywords:** acute heart failure (ahf), bnp, ef: ejection fraction, nt-pro bnp, nyha class

## Abstract

Background

Among the various biomarkers, B-type natriuretic peptide (BNP) and N-terminal pro-BNP (NT-proBNP) have proved particularly useful in helping clinicians diagnose acute heart failure (AHF) and evaluate the risk a patient faces at the time of presentation. However, data from the Indian subcontinent, particularly from the Kashmir Valley, with its unique demographic profile and altitude-related cardiovascular burden, remain sparse. This study aimed to compare the utility of these biomarkers in the management of AHF in a tertiary care setting and to evaluate their relationship with disease severity parameters, including New York Heart Association (NYHA) functional class, ejection fraction, and lung ultrasound B-lines.

Methods

The study was a prospective observational study conducted at the Government Medical College, Srinagar, over a period of 18 months. A total of 223 patients admitted to the hospital with acute heart failure were included. Both BNP and NT-proBNP were measured simultaneously at admission. All patients underwent lung ultrasonography (LUS) for B-line quantification before and after diuretic therapy and echocardiography for ejection fraction assessment. Statistical analyses included Pearson and Spearman correlations, one-way ANOVA, paired t-tests, and receiver operating characteristic (ROC) curve analysis.

Results

The majority of patients were male, 133 (59.6%), with a mean age of 62.3 ± 10.7 years. Mean BNP and NT-proBNP levels were 581.5 ± 180.1 pg/mL and 1707.5 ± 629.5 pg/mL, respectively. The two biomarkers correlated strongly with each other (Pearson r = 0.750, p < 0.001), and both varied significantly across NYHA functional classes (BNP: F = 6.118, p = 0.001; NT-proBNP: F = 4.405, p = 0.005).

Lung ultrasound B-lines showed a significant reduction following diuretic therapy (15.24 ± 1.07 vs 6.77 ± 1.06; p < 0.001). NT-proBNP showed non-significantly higher diagnostic performance over BNP for severe HF (area under the curve (AUC) 0.420 vs. 0.387). No significant difference was noted between the two biomarkers with respect to ejection fraction categories.

Conclusion

Both BNP and NT-proBNP are comparably effective in reflecting the haemodynamic and clinical severity of acute heart failure in the Kashmiri population. Their strong mutual correlation supports interchangeable use in clinical practice. Integration of lung ultrasound B-line monitoring with natriuretic peptide measurement provides superior assessment of decongestion. These findings have direct implications for resource-limited tertiary care settings, where single-biomarker assay selection is often dictated by cost and availability.

## Introduction

Acute heart failure (AHF) is one of the most common causes of emergency hospitalisations across the globe and carries significant short- and long-term morbidity and mortality [1]. In India, the burden of heart failure is exacerbated by a high prevalence of hypertension, rheumatic heart disease, ischaemic heart disease, and diabetes mellitus, frequently affecting younger patients compared to their Western counterparts [2]. The Kashmir Valley, lying at roughly 1,600 metres above sea level, offers a distinct cardiovascular setting where altitude-driven haemodynamic changes combined with a notably high prevalence of valvular heart disease together shape the regional profile of acute heart failure [3].

Natriuretic peptides, specifically B-type natriuretic peptide (BNP) and N-terminal pro-B-type natriuretic peptide (NT-proBNP), have transformed the diagnostic and prognostic evaluation of heart failure over the past two decades. BNP is a biologically active hormone secreted by ventricular cardiomyocytes in response to increased wall stress, while NT-proBNP is the biologically inert N-terminal fragment of the prohormone, with a longer half-life and greater plasma stability [4-5]. Both markers have been incorporated into international guidelines for the diagnosis and management of AHF, with established diagnostic cutoff values: BNP levels exceeding 100 pg/mL and NT-proBNP levels above 300 pg/mL (or age-adjusted thresholds) being considered positive for heart failure in the appropriate clinical context [6-7].

Despite robust evidence from European and North American trials, comparative data from the Indian subcontinent remain limited, and data from Kashmir are sparse. The region's unique body composition, altitude physiology, comorbidity profiles, and healthcare access patterns mean that clinical practice must be based on evidence from the region. Furthermore, the relative cost burden of NT-proBNP versus BNP assays in resource-constrained settings often forces clinicians to choose one over the other, necessitating clarity on their comparative utility.

Lung ultrasonography (LUS) with B-line quantification has emerged as a reliable bedside tool for extravascular lung water and tracking the efficacy of decongestion therapy in acute heart failure (AHF) [8]. Its integration with natriuretic peptide measurement offers a multi-modal approach to managing AHF that is practical even in emergency settings. However, the comparative performance of BNP versus NT-proBNP, contextualised against LUS parameters, has not been formally investigated in a Kashmiri cohort.

This study was therefore designed to compare the baseline levels and correlation of BNP and NT-proBNP in hospitalised AHF patients; evaluate their relationship with clinical severity parameters, including NYHA functional class, ejection fraction, and chest X-ray (CXR) congestion; assess their correlation with LUS B lines before and after diuretic therapy; and determine their respective diagnostic performances for identifying severe AHF in a Kashmiri tertiary care population.

## Materials and methods

Study design and setting

The study was a prospective, observational, single-centre study conducted in the Department of Internal Medicine of the Government Medical College (GMC) Srinagar. The study was conducted over a period of 18 months. GMC Srinagar is the premier tertiary care and teaching institution of the Kashmir Valley, receiving patients from the entire valley and adjoining districts of the Jammu and Kashmir Union Territory.

Patient selection

Adult patients (≥18 years) admitted to the emergency department or medicine ward with a primary diagnosis of acute heart failure were considered for enrolment. AHF was defined according to the 2021 European Society of Cardiology (ESC) Guidelines for the Diagnosis and Treatment of Acute and Chronic Heart Failure, incorporating clinical, radiological, and echocardiographic criteria [1-9]. Patients with a concurrent acute respiratory illness confounding the clinical assessment (e.g., severe pneumonia with septic shock); renal failure with estimated glomerular filtration rate (eGFR) <15 mL/min/1.73 m², as NT-proBNP levels are substantially confounded by renal function; patients with acute coronary syndrome as the primary admission diagnosis; those who refused consent; and those with incomplete data were excluded from the final analysis.

Ethical clearance

The study was reviewed and approved by the Institutional Review Board of Government Medical College Srinagar (Approval No. IRB/GMC-SGR/Med/567). Informed written consent was obtained from every participant or their legally designated representative before enrolment, and the study was conducted in accordance with the Declaration of Helsinki.

Data collection and clinical assessment

A pre-structured proforma was used to record demographic details, presenting symptoms, comorbidities, New York Heart Association (NYHA) functional class at admission, and relevant physical examination findings [7-20]. NYHA classification was assigned by the admitting physician, who was blinded to the biomarker results. Hypertension, diabetes mellitus, coronary artery disease (CAD), chronic atrial fibrillation (AF), hypothyroidism, and chronic obstructive pulmonary disease (COPD) were all carefully recorded.

Biomarker assays

Venous blood samples were drawn within two hours of admission, before initiation of diuretic therapy. BNP was measured using a fluorescence immunoassay on the Triage BNP system (Beckman Coulter, Brea, USA). NT-proBNP was measured using an electrochemiluminescence immunoassay on the Elecsys platform (Roche Diagnostics, Mannheim, Germany). Both assays were performed simultaneously on the same blood draw to ensure comparability.

Echocardiography

All patients underwent transthoracic echocardiography within 24 hours of admission, performed by a trained cardiologist following standard American Society of Echocardiography guidelines. Left ventricular ejection fraction (LVEF) was assessed using the modified biplane Simpson's method, and patients were subsequently categorised into three groups: heart failure with reduced ejection fraction (HFrEF; LVEF <40%), heart failure with mildly reduced ejection fraction (HFmrEF; LVEF 41-49%), and heart failure with preserved ejection fraction (HFpEF; LVEF ≥50%) [7-9].

Lung ultrasonography

Bedside lung ultrasonography was performed using a portable ultrasound machine (e.g., M7, Mindray, Shenzhen, China) with a 3.5 MHz curvilinear probe. B-lines were quantified in a four-zone protocol (right and left anterior and lateral chest zones). Each zone was assessed for the maximum number of B-lines visible in a single frame. The sum of B-lines across all zones constituted the LUS score (LUS-1 = pre-treatment, LUS-2 = post-diuretic therapy at 48-72 hours). The B-lines were identified as sharp, vertical, hyperechoic reverberation artefacts originating from the pleural line, extending to the far field without fading, and moving in synchrony with lung sliding. [8-17]

Chest radiography

A portable or standing posteroanterior (PA) chest radiograph was obtained for all patients at admission and assessed for radiological features of pulmonary congestion, including cardiomegaly, pulmonary vascular redistribution, interstitial oedema, alveolar opacification, and pleural effusion.

Statistical analysis

All statistical analyses were carried out using R (version 4.x; R Foundation for Statistical Computing, Vienna, Austria) and Python (Python Software Foundation, Wilmington, USA), incorporating libraries including *pandas*, *scipy*, *statsmodels*, and *scikit-learn*. Continuous variables were presented as mean ± SD or median with interquartile range, while categorical variables were expressed as frequencies and percentages. Normality was evaluated using the Kolmogorov-Smirnov test. Linear relationships were examined through Pearson or Spearman correlation coefficients, depending on data distribution. Differences across NYHA classes and ejection fraction categories were analysed using a one-way ANOVA, followed by Tukey's honestly significant difference (HSD) post hoc test, while sex-based comparisons were made using an independent sample t-test. A paired t-test was used to compare B-line counts prior to and following diuretic therapy. Receiver operating characteristic (ROC) curves were generated to assess the diagnostic accuracy of BNP and NT-proBNP in identifying severe heart failure (NYHA III/IV). A p-value below 0.05 was regarded as statistically significant.

## Results

Baseline demographic and clinical characteristics

The study enrolled a total of 223 patients with confirmed AHF. The mean age was 62.3 ± 10.7 years (range: 38-80). Male patients constituted 133 (59.6%) of the cohort, with female patients comprising 90 (40.4%). Table 1 summarises the baseline demographics and clinical characteristics.

**Table 1:** Baseline Demographic and Clinical Characteristics (N=223) Values are presented as mean ± standard deviation (SD) or number and percentage (n), as appropriate. SD: standard deviation; n: number; NYHA: New York Heart Association; HFrEF: heart failure with reduced ejection fraction; HFmrEF: heart failure with mildly reduced ejection fraction; HFpEF: heart failure with preserved ejection fraction; LVEF: left ventricular ejection fraction; CXR: chest X-ray.

Parameter	Value
Total Patients	223
Age, Mean ± SD (years)	62.3 ± 10.7
Age Range (years)	38 – 80
Male, n (%)	133 (59.6%)
Female, n (%)	90 (40.4%)
NYHA Class I, n (%)	15 (6.7%)
NYHA Class II, n (%)	53 (23.8%)
NYHA Class III, n (%)	121 (54.3%)
NYHA Class IV, n (%)	34 (15.2%)
HFrEF (LVEF <40%), n (%)	49 (22.0%)
HFmrEF (LVEF 41-49%), n (%)	50 (22.4%)
HFpEF (LVEF ≥50%), n (%)	124 (55.6%)
Mean LVEF ± SD (%)	51.5 ± 11.0
CXR Pulmonary Congestion, n (%)	146 (65.5%)

Comorbidity profile

Hypertension was the most common comorbidity, affecting 211 patients (94.5%), followed by chronic atrial fibrillation in 139 (62.5%), coronary artery disease in 125 (56%), diabetes mellitus in 94 (42%), hypothyroidism in 31 (13.5%), and other conditions in 26 (11.5%). Clinically, breathlessness was universal, present in all 223 patients (100%), while palpitations were reported in 175 (78.5%), fatigue in 157 (70.5%), cough in 143 (64.5%), and pedal oedema in 87 (39%).

Natriuretic peptide levels

The mean BNP at admission was 581.5 ± 180.1 pg/mL (median: 560.0 pg/mL, range: 212-1262 pg/mL). The mean NT-proBNP was 1707.5 ± 629.5 pg/mL (median 1603.0 pg/mL, range: 586-4880 pg/mL). All 223 patients had BNP above the diagnostic cutoff of 100 pg/mL; 198 (88.8%) had BNP >400 pg/mL. All patients had NT-proBNP above 300 pg/mL; 211 (94.6%) had NT-proBNP >900 pg/mL (Table 2).

**Table 2:** Distribution of Natriuretic Peptide Levels Values are presented as mean ± standard deviation (SD) or median with interquartile range (IQR). Diagnostic thresholds for BNP (<100, 100–400, >400 pg/mL) and NT-proBNP (<300, 300–900, >900 pg/mL) were applied per established heart failure guidelines. BNP and NT-proBNP showed a strong positive correlation (Pearson r = 0.750, p < 0.001). SD: standard deviation; IQR: interquartile range; BNP: B-type natriuretic peptide; NT-proBNP: N-terminal pro-B-type natriuretic peptide; n: number.

Parameter	BNP (pg/mL)	NT-proBNP (pg/mL)
Mean ± SD	581.5 ± 180.1	1707.5 ± 629.5
Median (IQR)	560.0 (460-680)	1603.0 (1214-2100)
Range	212 – 1262	586 – 4880
<100 / <300 pg/mL, n (%)	0 (0%)	0 (0%)
100-400 / 300-900 pg/mL, n (%)	25 (11.2%)	12 (5.4%)
>400 / >900 pg/mL, n (%)	198 (88.8%)	211 (94.6%)

Correlation between BNP and NT-proBNP

**Figure 1:**
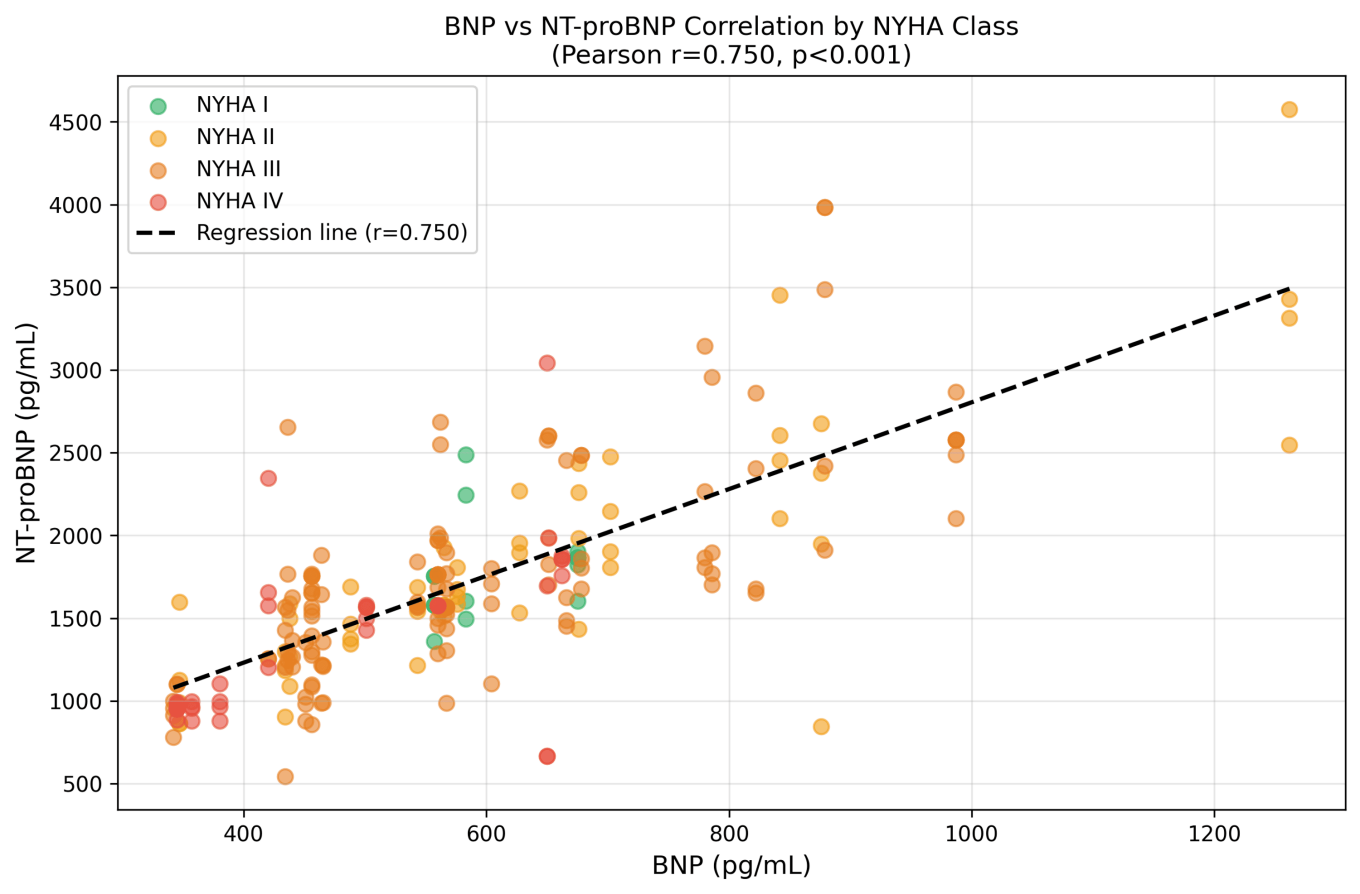
Correlation between BNP and NT-proBNP across NYHA functional classes. Each data point represents one patient, color-coded by NYHA class. The dashed black line represents the overall regression line (r = 0.750, p < 0.001). BNP: B-type natriuretic peptide; NT-proBNP: N-terminal pro-B-type natriuretic peptide; NYHA: New York Heart Association

A strong, statistically significant positive correlation was found between BNP and NT-proBNP levels (Pearson r = 0.750, p < 0.001). This correlation persisted across all NYHA functional classes (Figure 1). The regression equation for NT-proBNP from BNP was NT-proBNP = 2.618 × BNP + 184.7 (R² = 0.562).

Biomarker levels by NYHA functional class

**Figure 2:**
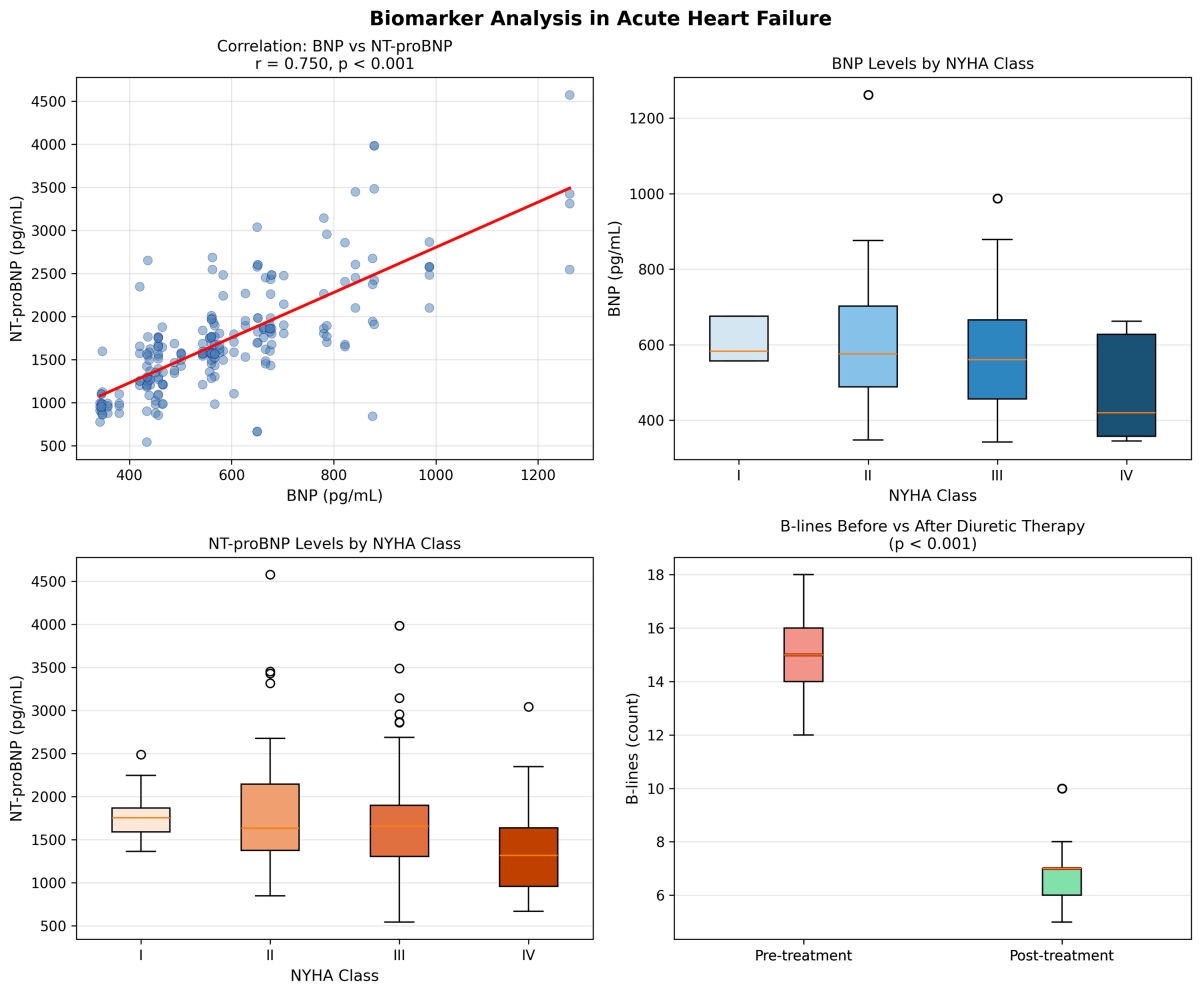
Box-plot comparison of BNP and NT-proBNP levels across NYHA functional classes (I-IV). Progressive trend is noted with peak levels in NYHA Class II and a reduction in Class IV, consistent with neurohormonal exhaustion in end-stage heart failure. Horizontal lines within boxes represent medians; boxes represent IQR; whiskers extend to 1.5 × IQR. BNP: B-type natriuretic peptide; NT-proBNP: N-terminal pro-B-type natriuretic peptide; NYHA: New York Heart Association

Both BNP and NT-proBNP showed significant differences across NYHA classes (Table 3). One-way ANOVA demonstrated a statistically significant overall difference for BNP (F = 6.118, p = 0.001) and NT-proBNP (F = 4.405, p = 0.005) across NYHA Classes I through IV. Notably, mean BNP was lower in NYHA Class IV (477.1 ± 125.1 pg/mL) compared to Class II (638.7 ± 235.6 pg/mL). This paradoxical finding is consistent with previously described mechanisms in end-stage heart failure: (i) terminal neurohormonal exhaustion, wherein failing cardiomyocytes progressively lose their synthetic capacity for natriuretic peptides [14]; (ii) reduced ventricular wall stress in extremely low cardiac output states, which diminishes the primary stimulus for BNP secretion; (iii) cardiac cachexia and reduced muscle mass prevalent in NYHA Class IV patients, leading to lower absolute peptide synthesis; and (iv) the relatively small sample size of the NYHA IV subgroup (n=34) in this cohort, which may have contributed to greater variability and a lower observed mean (Figure 2).

**Table 3:** Natriuretic peptide levels by NYHA functional class BNP: B-type natriuretic peptide; NT-proBNP: N-terminal pro-B-type natriuretic peptide; NYHA: New York Heart Association

NYHA Class	n	Mean BNP ± SD (pg/mL)	Mean NT-proBNP ± SD (pg/mL)
I	15	611.1 ± 55.0	1785.6 ± 287.2
II	53	638.7 ± 235.6	1832.0 ± 729.4
III	121	582.2 ± 163.4	1739.4 ± 614.3
IV	34	477.1 ± 125.1	1365.8 ± 518.2
p-value (ANOVA)	-	0.001	0.005

Biomarker levels by ejection fraction category

Table 4 presents the mean BNP and NT-proBNP levels stratified by ejection fraction category. Patients with HFrEF had numerically higher mean values of both biomarkers compared to HFmrEF and HFpEF, though neither difference reached statistical significance on ANOVA (BNP: F = 1.093, p = 0.337; NT-proBNP: F = 2.364, p = 0.096). This finding may reflect the heterogeneity of haemodynamic derangement across ejection fraction (EF) strata

**Table 4:** Natriuretic peptide levels by ejection fraction category EF: ejection fraction; HFrEF: heart failure with reduced ejection fraction; HFmrEF: heart failure with mildly reduced ejection fraction; HFpEF: heart failure with preserved ejection fraction; BNP: B-type natriuretic peptide; NT-proBNP: N-terminal pro-B-type natriuretic peptide

EF Category	n	Mean BNP ± SD (pg/mL)	Mean NT-proBNP ± SD (pg/mL)
HFrEF (<40%)	49	615.1 ± 224.5	1809.0 ± 790.8
HFmrEF (41-49%)	50	570.4 ± 176.1	1810.0 ± 706.6
HFpEF (≥50%)	124	572.8 ± 161.2	1626.1 ± 507.1
p-value (ANOVA)	-	0.337 (NS)	0.096 (NS)

Sex-based differences in biomarker levels

Male patients had significantly lower BNP levels compared to females (548.7 ± 127.1 vs. 630.1 ± 230.2 pg/mL; p = 0.001). NT-proBNP was also lower in males, but the difference did not reach statistical significance (1651.3 ± 573.8 vs. 1790.6 ± 698.8 pg/mL; p = 0.105).

Lung ultrasound B-lines and response to therapy

The mean pre-treatment B-line score (LUS-1) was 15.24 ± 1.07, which decreased significantly to 6.77 ± 1.06 post-diuretic therapy (LUS-2). The difference was statistically highly significant on the paired t-test (t = 116.99, p < 0.001), reflecting effective decongestion. The percentage reduction in B-lines was 55.6%, underscoring the sensitivity of LUS in monitoring therapeutic response (Table 5).

**Table 5:** B-line scores before and after diuretic therapy LUS: lung ultrasound

Parameter	Pre-treatment (LUS-1)	Post-treatment (LUS-2)	p-value
Mean B-lines ± SD	15.24 ± 1.07	6.77 ± 1.06	<0.001
95% CI	15.10 – 15.38	6.63 – 6.91	-
Range	12 – 18	5 – 10	-

Receiver operating characteristic (ROC) curve analysis

**Figure 3:**
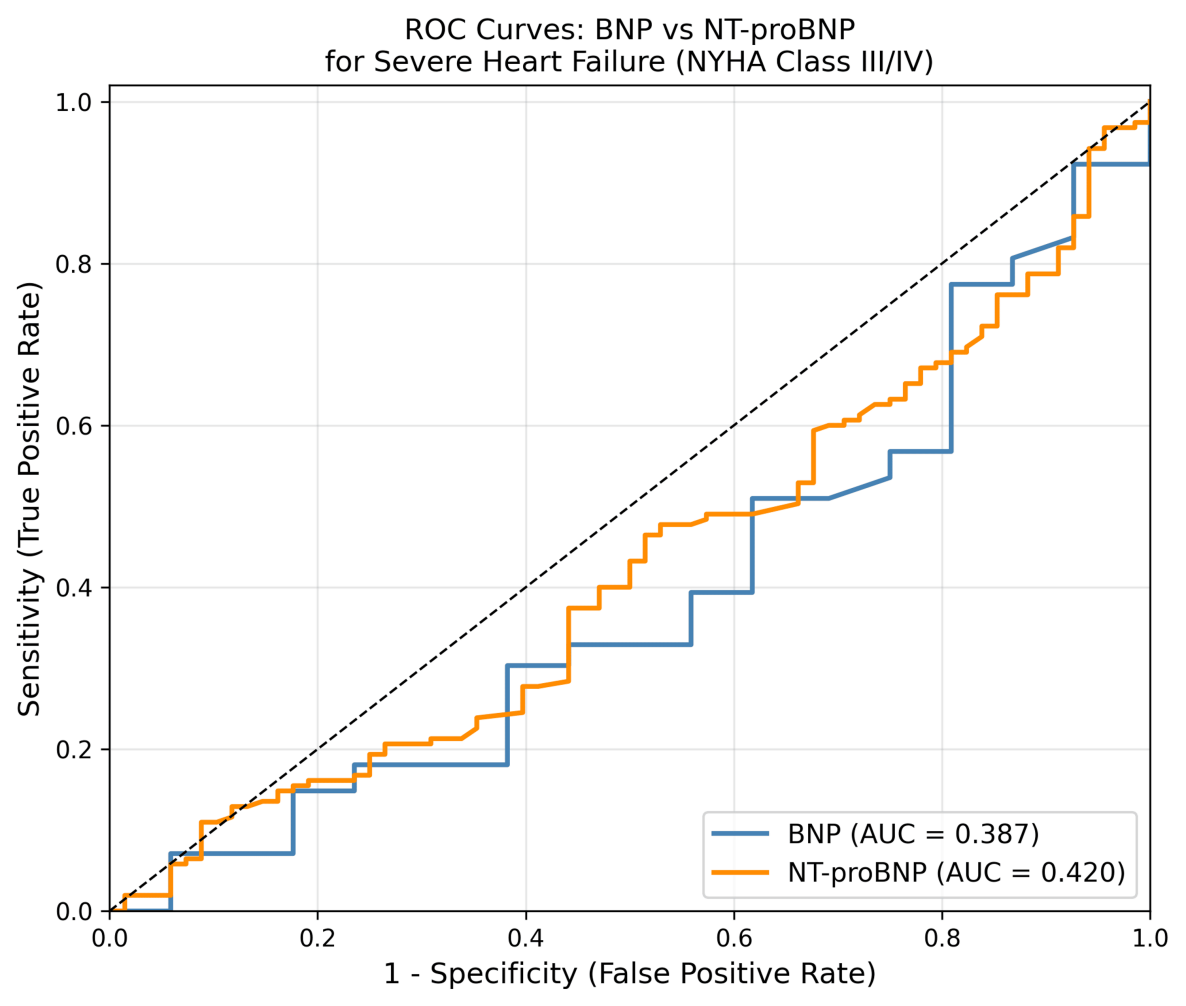
Receiver operating characteristic (ROC) curves for BNP and NT-proBNP in discriminating severe acute heart failure (NYHA Class III/IV). The area under the ROC curve (area under the curve (AUC)) for BNP was 0.387, and for NT-proBNP was 0.420, indicating poor discriminative performance within this uniformly elevated AHF cohort. BNP: B-type natriuretic peptide; NT-proBNP: N-terminal pro-B-type natriuretic peptide; NYHA: New York Heart Association; AHF: acute heart failure

Receiver operating characteristic curve analysis for discriminating severe AHF (NYHA Class III/IV) from less severe forms yielded an AUC of 0.387 (95% CI: 0.31-0.46) for BNP and 0.420 (95% CI: 0.34-0.50) for NT-proBNP. The modest AUC values reflect the fact that both biomarkers are elevated across the entire AHF spectrum in this cohort, and both Class I-II and Class III-IV patients showed markedly elevated values, reducing the discriminative power between severity strata (Figure 3). These AUC values (BNP: 0.387; NT-proBNP: 0.420) indicate poor discriminatory ability for differentiating severity strata within an established AHF cohort and should not be interpreted as reflecting diagnostic failure of the biomarkers per se, but rather as the expected limitation of within-AHF severity discrimination in a uniformly elevated biomarker population.

## Discussion

This study offers the most comprehensive direct comparison of BNP and NT-proBNP conducted exclusively within an acute heart failure cohort drawn from the Kashmiri population of northern India. The key findings of the study are (i) both biomarkers are markedly elevated in AHF patients from this region; (ii) there is a strong mutual correlation (r = 0.750), supporting interchangeable clinical utility; (iii) both markers reflect symptom severity as measured by NYHA class; (iv) neither marker significantly discriminates AHF by ejection fraction phenotype; and (v) LUS B-lines provide a sensitive measure of decongestion that complements natriuretic peptide assessment.

The demographic characteristics of our cohort, including a mean age of 62.3 years, male predominance, and high prevalence of hypertension in 211 (94.5%), atrial fibrillation in 139 (62.5%), and coronary artery disease in 125 (56%), are broadly consistent with previously reported Indian heart failure cohorts, where patients tend to present at a younger age compared to Western counterparts [10]. The unusually high comorbidity burden observed in our cohort likely reflects the tertiary care referral pattern, which serves as the premier referral institution for the entire Valley. Patients with advanced multi-morbidity and complex cardiovascular profiles are preferentially referred to this centre, resulting in a cohort that is not representative of the general community AHF population but rather of a high-acuity, multi-morbid subset. This referral bias should be considered when interpreting comorbidity prevalence in this study. The predominance of HFpEF in 124 patients (55.6%) over HFrEF in 49 (22%) reflects emerging epidemiological patterns across South Asia, where hypertensive cardiomyopathy and valvular disease account for a substantial proportion of heart failure cases [11].

The strong correlation between BNP and NT-proBNP observed in this study is consistent with earlier published literature. Fonseca C et al. demonstrated excellent concordance between BNP and NT-proBNP in patients presenting with dyspnoea [12]. The slightly lower correlation in our cohort may reflect population-specific differences in renal function, body mass index, and the HF phenotype distribution. The longer plasma half-life of NT-proBNP (60-120 minutes vs. 20 minutes for BNP) makes it more susceptible to cumulative elevation in patients with repeated admissions, a pattern commonly seen in Kashmir, where patients often present late due to geographic access barriers [13].

The significant variation in both biomarker levels across NYHA classes confirms that both markers reflect neurohormonal activation, which is proportional to functional impairment. The paradoxical reduction in biomarker levels in NYHA Class IV compared to Class II, observed in our cohort, has been previously described and attributed to the phenomenon of terminal neurohormonal exhaustion in end-stage HF, wherein failing cardiomyocytes lose their synthetic capacity [14]. Furthermore, the small NYHA IV subgroup (n=34) and the presence of cardiac cachexia, commonly seen in advanced HF patients referred to tertiary centres, may have contributed to the lower observed BNP values in this severity stratum. Clinicians should therefore be cautious about relying solely on absolute BNP levels to grade severity in NYHA Class IV patients. Additionally, the extremely low cardiac output states in Class IV may limit the ventricular wall stress that serves as the primary stimulus for natriuretic peptide secretion.

The absence of a statistically significant difference in biomarker levels across ejection fraction categories is an important and clinically relevant finding. Theoretically, one might expect that HFrEF, with its greater systolic dysfunction and higher wall stress, would produce higher natriuretic peptide levels. Our findings align with the existing literature showing that natriuretic peptide levels maintain their diagnostic and prognostic value across the full spectrum of ejection fraction categories; however, they cannot, on their own, reliably differentiate HFrEF from HFpEF, given that considerable neurohormonal activation may equally be present in patients with HFpEF. [15-16] This has significant therapeutic implications, as NT-proBNP-guided therapy is recommended in HFrEF guidelines, and clinicians should be cautious about using biomarker levels alone to determine EF phenotype.

The marked and statistically significant reduction in LUS B-lines following diuretic therapy (55.6% reduction, p < 0.001) provides strong validation for bedside LUS as a monitoring tool in AHF management. This finding is consistent with the landmark LUS-HF trial and subsequent meta-analyses, which confirmed that B-line-guided diuresis reduces rehospitalisation rates [17]. The integration of LUS with natriuretic peptide measurement provides a multimodal, bedside-accessible approach to AHF management that is particularly valuable in settings like GMC Srinagar, where advanced haemodynamic monitoring facilities are not always available.

The modest AUC values for both biomarkers in discriminating severe from less severe AHF (0.387 for BNP, 0.420 for NT-proBNP) deserve interpretation in context. All patients in this cohort had confirmed AHF with uniformly elevated biomarker levels, meaning the ROC analysis was essentially discriminating within a uniformly elevated AHF population rather than distinguishing AHF from non-AHF. In such a scenario, the overlap of biomarker levels across severity strata is expected, as both Class I-II and Class III-IV patients share substantially elevated values. It must be explicitly acknowledged that both biomarkers demonstrated poor discriminatory ability (AUC < 0.5) for within-cohort severity stratification, which is an expected finding when ROC analysis is applied to a population uniformly fulfilling AHF diagnostic criteria. The marginally superior AUC of NT-proBNP for severe HF is consistent with its established role as a superior prognostic marker, given its longer half-life and greater plasma stability [18].

The sex-based difference in BNP levels (females > males, p = 0.001) has been well described in the literature and is attributed to the modulatory effects of sex hormones on natriuretic peptide secretion; oestrogen upregulates BNP expression, while testosterone has an inhibitory effect [19]. Interestingly, this difference was not statistically significant for NT-proBNP in our cohort, which may reflect the influence of renal clearance differences between sexes on the longer-lived NT-proBNP molecule.

From a clinical practice standpoint, this study supports the use of either BNP or NT-proBNP as equally valid first-line biomarkers in AHF management in this region. In resource-limited settings, where an assay is not routinely available, cost, turnaround time, and laboratory infrastructure may reasonably guide the choice. The combination of either biomarker with LUS provides a robust, low-cost, radiation-free approach to AHF monitoring that should be standardised in tertiary care hospitals across Jammu and Kashmir.

This study carries certain limitations worth acknowledging. Being a single-centre study, the findings may not be fully generalisable to the broader Indian population. The lack of a non-AHF comparator group restricts formal diagnostic sensitivity and specificity calculations. Additionally, serial natriuretic peptide measurements during hospitalisation were not obtained, preventing an assessment of in-hospital biomarker trends. The four-zone lung ultrasound protocol, though practically convenient, may have underestimated B-line counts relative to more exhaustive eight- or twenty-eight-zone approaches. Lastly, long-term outcomes, including rehospitalisation rates and mortality, were not captured, limiting conclusions regarding prognostic value. Multivariable regression analysis was not performed in this study. Given the observational, descriptive design primarily aimed at comparing biomarker utility rather than identifying independent predictors. Future studies with larger sample sizes and defined outcome endpoints would be better suited to explore independent predictors of AHF severity in this population.

## Conclusions

In patients admitted with acute heart failure in a Kashmiri tertiary care setting, BNP and NT-proBNP demonstrate a strong mutual correlation and equivalent utility in reflecting clinical and haemodynamic severity. Both biomarkers show significant variation across NYHA functional classes but do not reliably differentiate AHF by ejection fraction phenotype. Both biomarkers demonstrated poor discriminatory ability for within-cohort severity stratification, consistent with the expected limitation of ROC analysis in a uniformly elevated AHF population. Lung ultrasound B-line monitoring provides an effective and sensitive adjunct to natriuretic peptide measurement for guiding decongestion therapy. Region-specific evidence, such as these findings, is essential for informing evidence-based AHF management protocols in Jammu and Kashmir.
